# CIRSE Standards of Practice for the Use of Non-sclerosant Liquid Embolic Agents in IR Practice

**DOI:** 10.1007/s00270-026-04610-4

**Published:** 2026-09-23

**Authors:** Francesco Giurazza, Anna Maria Ierardi, José Urbano, Clare Bent, Pietro Roccatagliata, Romaric Loffroy

**Affiliations:** 1 Vascular and Interventional Radiology Department, Cardarelli Hospital, Naples, Italy; 2 Fondazione IRCCS Ca’ Granda Ospedale Maggiore Policlinico, University of Milan, Milan, Italy; 3 Vascular and Interventional Radiology, Instituto Ramón y Cajal de Investigación Sanitaria (IRYCIS), Ramón y Cajal University Hospital, Madrid, Spain; 4 University Hospitals Dorset, Bournemouth, Poole, UK; 5 Cardarelli Hospital, Naples, Italy; 6 Department of Vascular and Interventional Radiology, Université Bourgogne Europe, François-Mitterrand University Hospital, Dijon, France

**Keywords:** Liquid embolics, Embolization, Endovascular, Glue, Cyanoacrylate, Polymers, Non-sclerosant

## Abstract

**Background:**

Non-sclerosant liquid embolic agents are safe and effective embolics that have been used in interventional radiology for more than 50 years, in both emergency and elective scenarios.

**Purpose:**

This document aims to review the chemical principles and technical considerations involved, to make recommendations regarding clinical indications for the use of non-sclerosant liquid embolic agents, and to review the literature regarding outcomes and complications. CIRSE Standards of Practice documents are not intended to impose a standard of clinical patient care, but to recommend a reasonable approach to, and best practices for, procedural performance, in this instance the use of non-sclerosant liquid embolic agents.

**Methods:**

The writing group was established by the CIRSE Standards of Practice Committee and consisted of five clinicians with internationally recognised expertise in non-sclerosant liquid embolic agents and a research assistant (P.R.). The writing group reviewed the existing literature on non-sclerosant liquid embolic agents, performing a pragmatic evidence search using PUBMED and EMBASE focussing on English-language publications, preferably from 2009 up to December 2025, and including more than ten patients. Where clear gaps were evident, older literature was searched and select references included. The final recommendations were formulated through consensus.

**Results:**

Non-sclerosant liquid embolic agents are represented by cyanoacrylates and polymers which are safe and effective agents that can be used in a wide range of clinical scenarios, resulting in effective embolization even in patients with impaired coagulation function. Knowing their chemical properties provides understanding of their correct preparation, technical application and clinical indication. Compared to mechanical and particulate agents, non-sclerosant liquid embolic agents present a steeper learning curve, and thus, thorough training in their use is essential.

## Introduction

The CIRSE Standards of Practice Committee established a writing group tasked with developing up-to-date recommendations for embolization procedures using non-sclerosant liquid embolic agents (NSLEA). Neuroradiological endovascular interventions were intentionally excluded from the scope of this document. It is important to note that CIRSE Standards of Practice documents are neither clinical practice guidelines nor systematic literature reviews. They are designed to outline reasonable approaches and best practices, providing interventional radiologists with practical guidance while allowing flexibility in patient care.

Institutions are encouraged to review their internal procedures regularly, with an emphasis on quality improvement and ongoing professional development. Recommendations should be considered alongside international guidance, local resources, and findings from routine internal morbidity and mortality reviews.

Key recommendations for the use of NSLEA in endovascular interventions are summarised in Table 1, highlighting their versatility, permanence and technique considerations.

**Table 1:** Key recommendations for the use of NSLEA in endovascular embolization interventions

Key recommendations
1. NSLEA are versatile agents suitable for emergent and elective interventions, particularly for use in small- and medium-sized vessels
2. Two main categories exist based on their biochemical behaviour: *adhesive* (cyanoacrylates, or glues): adhere to, and bond surrounding tissues *cohesive* (polymers): bond solely to themselves
3. Radiopaque additives are required for fluoroscopic visualisation of both cyanoacrylates and polymers
4. NSLEA produce permanent vascular occlusion, independent of coagulation status and are thus exceptionally useful in emergency settings or when temporary cessation of antiplatelet/anticoagulation therapies is not feasible
5. Both cyanoacrylates and polymers can be combined with mechanical embolics (e.g. coils or plugs), to broaden applicability in large calibre vessels with high-flow
6. NSLEA delivery should always be the final step if using a single catheter, as traces of liquid embolic adhering to the inner lumen of the catheter can compromise or prevent additional embolic delivery
7. The minimum dilution of cyanoacrylates with ethiodized oil should not be lower than 1:1 to avoid inadequate radiopacity and excessively rapid polymerisation before reaching the target; conversely, the maximum dilution should not exceed 1:8, as excessive ethiodized oil reduces the embolic effect of cyanoacrylate
8. Mixing of cyanoacrylate and ethiodized oil should only require 4–5 gentle transfers between syringes via a 3-way stopcock, and the mixture should remain clear. If the mixture turns white, polymerisation is occurring and the process should be re-started
9. When using cyanoacrylates, the time between dextrose and glue injections should be kept as brief as possible and back-flow of blood in the microcatheter should be prevented
10. Operators should consider the volume of preparatory dextrose injected: distal embolization targets require generous dextrose flushing to minimise contact between cyanoacrylate and blood anions, favouring glue propagation into small vessels; proximal embolization requires only microcatheter dead space flushing, aiming to obtain early polymerisation just after injection
11. If glue is used in slow flow vessels (e.g. venous malformations) or no-flow vessels (e.g. bile ducts) the amount of glucose should be minimised, because glucose is not rapidly transported away and it prevents polymerisation
12. When using polymers, once the microcatheter has been flushed with saline, it is advisable to leave a Luer-lock syringe connected to the hub of the microcatheter to prevent reflux of blood while the polymer is being prepared
13. When injected via catheter (as opposed to direct transcutaneous injection), both cyanoacrylates and polymers must be injected via microcatheters to ensure precisely controlled delivery; embolic injection using diagnostic catheters should be avoided
14. When injecting through a microcatheter of 2 Fr or less, using a 1 mL syringe offers a more controlled delivery
15. Performing test injections with iodinated contrast using a syringe that is identical to the one that will be then used for agent injection will enable the operator to estimate the volume to deliver and the injection speed
16. NSLEA have a steeper learning curve than mechanical or particulate embolics; thorough training in their use is essential to minimise complications

NSLEA: Non-sclerosant liquid embolic agents

## Methods

The writing group, established by the CIRSE Standards of Practice Committee, comprised five interventional radiologists (IRs) with internationally recognised expertise in liquid embolics, supported by a research assistant (P.R.). A pragmatic evidence search was performed on NSLEA using PUBMED and EMBASE, focussing on English-language publications, preferably from 2009 up to December 2025, and including more than ten patients; the main search terms included: liquid, embolics, embolization, endovascular, glue, cyanoacrylate, and polymers. Studies focussing on neuroradiological interventions and vein varicosities were not considered because they lie beyond the scope of the document. Reports lacking sufficient information on the characteristics of the liquid embolic used were also excluded. The final recommendations were developed through group consensus, integrating the available literature with expert experience.

## Background

Embolization is a minimally invasive technique that enables targeted occlusion of blood vessels through delivery of embolic agents via catheters, microcatheters, or needles. Embolic choice depends on vascular anatomy, clinical indication and patient-specific factors.

Embolic agents are customarily grouped into three categories according to their physical properties: mechanical occluders (such as coils and plugs), particulate agents (including microparticles, microspheres, and gelatin sponge), and liquid embolics (sclerosant and non-sclerosant).

Mechanical occluders and liquid embolics typically result in permanent vessel occlusion, whereas particulate agents can achieve either permanent or temporary occlusion depending on their composition. In many clinical scenarios, different embolic types are combined to exploit their complementary properties.

This document focuses on NSLEA currently available in Europe. The only guideline specific to cyanoacrylates, one type of NSLEA used in vascular embolization, was published in Japan in 2014 [1], highlighting the need for updated international recommendations.

## NSLEA

NSLEA have been used in interventional radiology for more than 50 years [2], primarily to occlude small- and medium-sized vessels. These agents have since undergone substantial development and are now categorised according to their biochemical behaviour:

- *Adhesive agents* (cyanoacrylates or ‘glues’), which adhere to and bond with surrounding tissues.
- *Cohesive agents* (polymers), which bond only to themselves.

Neither agent is intrinsically radiopaque and therefore requires the addition of radiopaque substances to enable visualisation under fluoroscopy. Both groups solidify on contact with blood, allowing effective embolization irrespective of coagulation status, including in patients receiving antiplatelet or anticoagulant therapy. Their specific characteristics are discussed in subsequent sections.

Not all cyanoacrylates hold formal approval for endovascular embolization, although off-label use is well documented. In contrast, polymer embolics were purpose-designed for endovascular treatments and are approved for this indication. The principal NSLEA available in Europe are listed in Table 2.

**Table 2:** List of the main NSLEA available in Europe

Category	Radiopacity induced by:
*Adhesive (Cyanoacrylate glues)*	
Glubran®2—GEM, Italy (approved for endovascular use in EU)	Ethiodized oil
MagicGlue®—Balt, France (approved for endovascular use in EU)	Ethiodized oil
Histoacryl®—B-Braun, Germany (not approved for endovascular use in EU)	Ethiodized oil
*Cohesive (EVOH-based polymers, all approved for endovascular use in EU)*	
Onyx®—Medtronic, US (shaking required)	Tantalum
Squid®—Balt, France (shaking required)	Tantalum
Menox®—Meril Life Science, India (shaking required)	Tantalum
Libro®—INVAMED, US (shaking required)	Tantalum
Lava®—NeuroSafe Medical, China (shaking required)	Tantalum
PHIL®—Terumo Neuro, US (shaking not required)	Iodine

EVOH: Ethylene vinyl alcohol; EU: Europe; US: United States of America

### Chemical Description

#### Cyanoacrylates

**Fig. 1:**
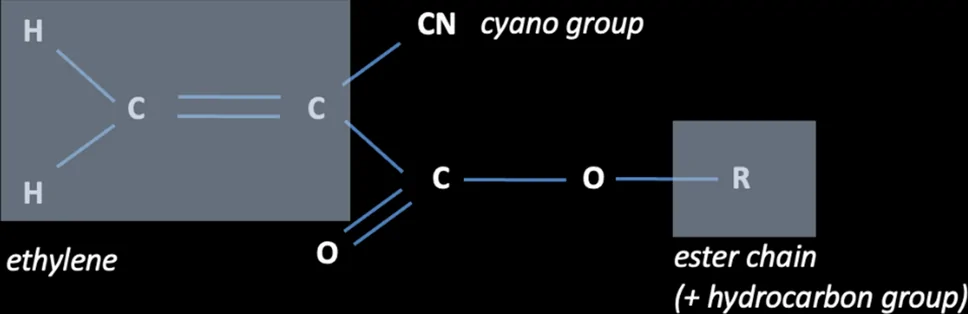
Chemical structure of cyanoacrylates

Cyanoacrylates used in embolization are fast-acting liquid adhesives derived from alkyl 2-cyanoacrylate monomers. In their liquid state, cyanoacrylates consist of monomer molecules which rapidly polymerise upon contact with water or anions, forming a solid intravascular cast. Variations in the number of hydrocarbons attached to the ester group determine the specific compound (Fig. 1), such as isobutyl cyanoacrylate and *N*-butyl cyanoacrylate (NBCA), and influence their chemical behaviour (Table 3). Lengthening of ester chains slows polymerisation, reduces heat generated during exothermic reaction and decreases cytotoxicity [3].

**Table 3:** Cyanoacrylates available in Europe for medical use: types and properties

	Polymerisation time	Adhesive strength	Tissue inflammation
Histoacryl®^*^ B-Braun, Germany* (NBCA)*	Fast	High	High
Glubran®2 GEM, Italy* (NBCA*+*MS)*	Intermediate	Intermediate	Intermediate
MagicGlue® Balt, France *(AHCA)*	Slow	Slow	Slow

NBCA: N-butyl cyanoacrylate; MS: Methacryloxysulfolane; AHCA: Alpha-hexyl cyanoacrylate

Grading is based on literature data [3, 5]

^*^Not approved for endovascular use

Cyanoacrylates have low viscosity and are easy to inject; however, they lack inherent radiopacity and polymerise immediately on contact with blood or anions, making direct injection impossible. An anion-free contrast medium must therefore be used to prepare the delivery system before administration. Ethiodized oil, an ethyl ester of iodinated poppy-seed oil fatty acids, is used for this purpose. When mixed with cyanoacrylate, ethiodized oil provides radiopacity and prolongs polymerisation time, allowing a more controlled, homogeneous solidification.

Cyanoacrylates should be refrigerated when stored, as they gradually polymerise at room temperature.

#### Polymers

**Fig. 2:**
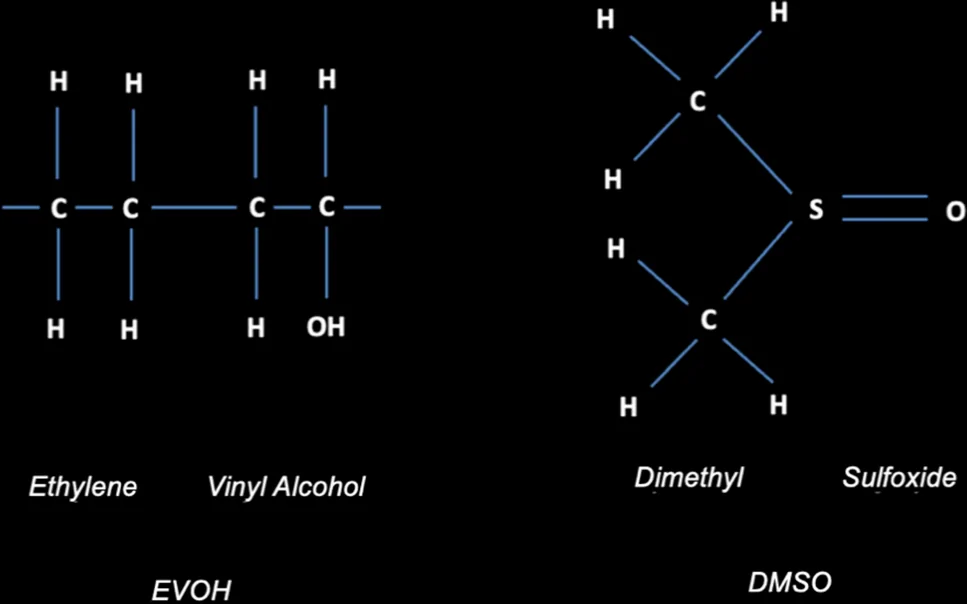
EVOH and DMSO chemical structure

Chemically, polymers are plastic-based compounds that remain in a liquid phase due to the solvent in which they are suspended, both during storage in the vial and during navigation within the microcatheter. In contrast to cyanoacrylates, they remain stable at room temperature, not requiring refrigeration. Once injected into the bloodstream, the solvent diffuses into the surrounding blood, causing the polymer to precipitate into a soft cohesive cast. The absence of chemical polymerisation and heat generation in this process underlies the distinctive non-adhesive behaviour of polymer-based embolic agents. The most widely used and extensively studied polymer is ethylene vinyl alcohol (EVOH). EVOH consists of long-chain molecules dissolved in dimethyl sulfoxide (DMSO), which acts as both a solvent and a carrier (Fig. 2). When DMSO diffuses into the bloodstream, the EVOH precipitates in a controlled manner, allowing the operator to build a cast with predictable morphology.

EVOH is available in multiple concentrations, typically between 4 and 8%, and marketed as EVOH 12, 18, and 34, reflecting their relative viscosity compared with saline. At higher concentrations, the formulation becomes more viscous, consequently reducing its capacity for distal penetration, enabling operators to tailor the embolization endpoint. More recently, additional polymers have been introduced, including polyhydroxyethylmethacrylate (pHEMA), a hydrophobic agent that also requires DMSO for delivery and is supplied in a range of viscosities (25%, 30%, and 35%).

Because EVOH lacks intrinsic radiopacity, additional elements must be incorporated to permit accurate fluoroscopic visualisation. The most common method involves incorporating Tantalum microparticles into the polymer matrix, which enhances radiopacity and facilitates clear visualisation during injection; however, these heavy particles sediment over time and therefore require vigorous agitation immediately before use to ensure a homogeneous mixture. A notable drawback of Tantalum-based systems is the permanent deposition of metallic particles within treated tissues, resulting in CT (computed tomography) artefacts that can impair post-procedural or oncologic imaging assessment, and tattooing, if the embolized vessels are very superficial. Also, Tantalum may cause injury during surgery in the embolized tissues due to high-energy electrical currents during electrocautery. To overcome these limitations, newer EVOH formulations incorporate Iodine directly into the polymer backbone, generating intrinsic radiopacity without the need for shaking. These agents eliminate the issue of sedimentation and reduce the degree of CT artefact while maintaining the handling characteristics of traditional EVOH systems.

### Mechanisms of Action

#### Cyanoacrylates

Cyanoacrylates undergo a rapid anionic polymerisation in weak bases (alkaline) like blood (pH 7.35–7.45). In this chain-growth reaction, double bonds are broken, resulting in the formation of strong, long polymer chains within seconds. The reaction is inhibited under acidic conditions. The adhesive polymer, composed of cyanoacrylate and blood, bonds firmly to the endothelium, reducing the risk of recanalisation. Polymerisation starts at the outer surface; if the amount of glue is very high and is forming a large ball, the inner cyanoacrylate may not have been in contact with a base and consequently not polymerised.

**Fig. 3:**
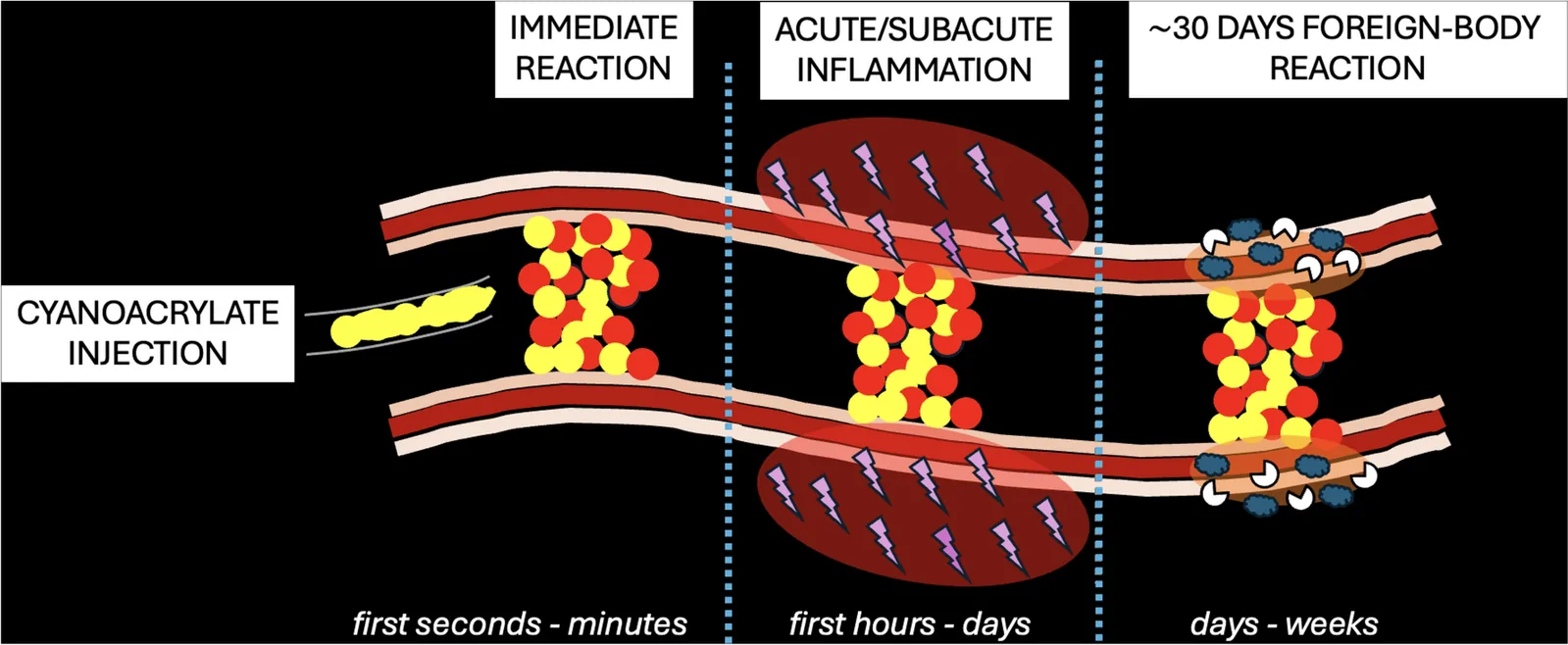
Mechanisms of action of cyanoacrylates. In the first few seconds after the injection of cyanoacrylates into blood vessels, polymerisation commences, including bonding with blood elements and adhering to the intima, finally inducing mechanical occlusion of blood flow. Between the first hours and days, vessel wall inflammation occurs, causing a foreign-body reaction characterised by macrophages, giant cells, and fibrosis, lasting for approximately one month

Beyond mechanical occlusion, cyanoacrylates elicit a range of tissue responses (Fig. 3). An acute inflammatory reaction within the intima and perivascular tissues evolves over approximately 30 days into a granulomatous foreign-body response. Key histological features include: intimal necrosis from direct cytotoxicity, perivascular oedema related to both exothermic polymerisation and delayed monomer toxicity, a foreign-body granulomatous reaction with slow degradation and incorporation into the vessel wall, occasional extravascular extrusion and capillary ingrowth within embolized vessels [3]. These processes promote durable vessel obliteration, albeit with associated local tissue injury.

Recent animal studies have provided insight into cyanoacrylate behaviour: blocked-flow injection produced more compact casts with less vascular disruption than free-flow injection [4]; medium-term follow-up showed persistent occlusion despite partial degradation, with granulomatous inflammation surrounding residual glue [5]. Clinically, cyanoacrylates provide durable occlusion superior to particulate agents, although rare recanalisation or migration events have been reported [6]. Cyanoacrylates are permanent embolic materials and not resorbed by the human body; however, over time, the ethiodized oil or added Iodine are resorbed, so the embolic material may become invisible on X-ray-based modalities.

#### Polymers

Polymers are non-adhesive, permanent liquid embolic agents, composed of EVOH or a hydrophobic polymer dissolved in anhydrous DMSO. Tantalum or Iodine is added for radiopacity. Viscosity depends on polymer concentration: lower concentrations are less viscous and allow greater distal penetration.

**Fig. 4:**
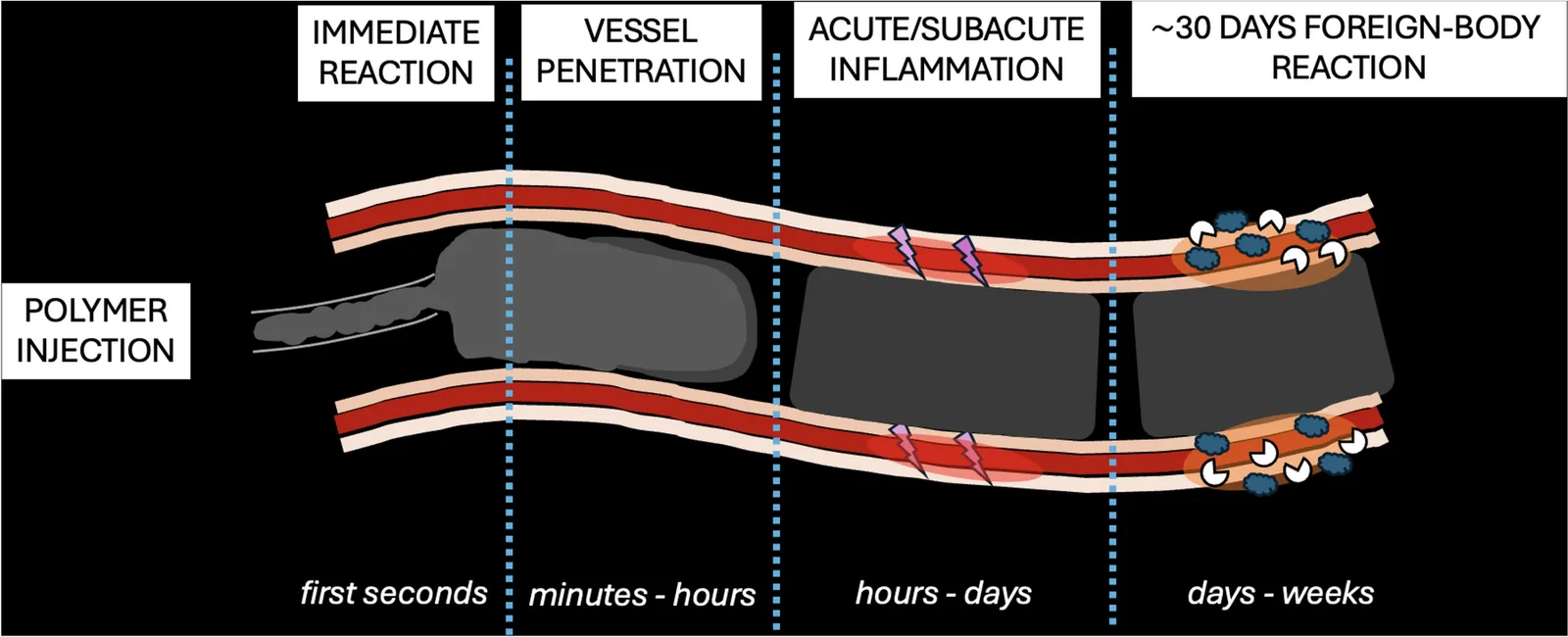
Mechanisms of action of polymers. In the first seconds after injection into blood vessels, polymers start to mechanically occlude blood flow via precipitation of the EVOH cast as DMSO dissolves. No adherence to blood components or vessel wall occurs. As the polymer injection continues, the cast propagates uniformly and distally into the vessel, the solidification process starting from the periphery of the cast and extending centrally. In the first days post-embolization, vascular inflammation occurs, resulting in a foreign-body reaction and vessel wall fibrosis in the first weeks

Upon injection into an aqueous environment, the EVOH/DMSO solution undergoes phase separation: DMSO rapidly diffuses into the surrounding fluid while EVOH precipitates, forming a soft, non-adherent polymer mass. Precipitation begins at the surface while the core remains liquid, allowing deep, ‘lava-like’ penetration within vessels without fragmentation (Fig. 4). The resulting rubbery EVOH cast conforms to the vessel architecture, mechanically occluding the lumen and providing immediate, durable occlusion, even in high-flow vessels; as with cyanoacrylates, this process is not influenced by coagulation status [7].

The EVOH cast’s non-adhesive property reduces the risk of catheter entrapment. Injection induces inflammation [8] and over time fibrosis and inflammatory encapsulation reinforce vessel occlusion.

## Technique

### Equipment Specifications

Liquid embolics may require preparation or be supplied ready-to-inject by the manufacturer. In either case, standard equipment should be used to ensure safe administration (Table 4).

**Table 4:** Equipment required for liquid embolization

Cyanoacrylates	Polymers
Ethiodized oil	Electric shaker (only for Tantalum-based polymers)
Metallic needles	Metallic needles
Microcatheters (compatible with ethiodized oil)	Microcatheters (compatible with DMSO)
Syringes (resistant to ethiodized oil)	Syringes (provided by manufacturers and compatible with DMSO)
3-way stopcock (resistant to ethiodized oil)	DMSO
Dextrose (G-5% or G-10%)	Saline

G-5%: Glucose 5%; G-10: % Glucose 10%; DMSO: Dimethyl sulfoxide

Cyanoacrylates are supplied in 0.5–1-mL vials and require angiography suite preparation [3]. The liquid is aspirated into a syringe via a metallic needle, while ethiodized oil (10 mL glass vial) should be aspirated using large-bore needles (14–18 G) or a mixing kwill due to its viscosity. Dilution can be performed using a three-way stopcock between syringes or in a metallic bowl before drawing up the intended volume into a syringe; in both cases, mixing via a three-way stopcock is advised in order to obtain the proper suspension. Plastic components in contact with ethiodized oil should be polyethylene or polypropylene to avoid degradation. Microcatheters or needles used for embolic delivery should be flushed with 5–10% dextrose prior to injection to displace anionic fluid (e.g. blood or contrast), in order to prevent premature polymerisation and catheter blockage.

Polymers are supplied in kits containing EVOH and DMSO vials [7]. Tantalum-based EVOH agents (e.g. Onyx® Medtronic, US; Squid® Balt, France) come in 1.5-mL or 6-mL vials and must be shaken before injection to suspend the Tantalum for radiopacity. Specific syringes are provided by the manufacturer, and metallic needles are required to aspirate the liquid. Hydrophobic polymers (e.g. PHIL® Terumo Neuro, US), which use Iodine for radiopacity, are supplied ready-to-inject in manufacturer-specific syringes and do not require shaking.

Both cyanoacrylates and polymers should be injected via microcatheters to ensure precise controlled delivery [3, 7]. Microcatheters initially developed for neurointerventional procedures are also used in peripheral embolization; detachable-tip or balloon microcatheters are particularly helpful for prolonged procedures or at target sites with a high risk of retrograde reflux.

Embolic injection using standard diagnostic catheters should be avoided due to proximal anatomic positioning, poor injection control due to catheter dead space and the risk of embolic adherence to the catheter tip, which could lead to non-target embolization if displaced during catheter removal. Microcatheters for polymers must be DMSO-compatible to avoid chemical degradation [7], while during cyanoacrylate injections, microcatheters need to be compatible with ethiodized oil.

For percutaneous approaches, a metallic needle is required, typically 18–21 G depending on lesion depth. Operators should note that both ethiodized oil and DMSO can damage needle hubs; when multiple injections are planned, replacing the needle reduces the risk of leakage.

Use of updated radiological imaging equipment is of paramount importance: NSLEA should be adopted with modern angiography suites providing high quality fluoroscopy with road-mapping functions (including reverse-roadmap) and cone-beam CT acquisitions to visualise embolics distribution upon injection.

### Liquid Preparation

#### Cyanoacrylates

Meticulous technique is essential for predictable outcomes with cyanoacrylates. They are used in combination with ethiodized oil to provide radiopacity and modulate polymerisation rate. The mixture ratio is determined by patient-specific and procedural factors, including vessel distance from the catheter tip, blood flow velocity, anatomical location, and intended embolization endpoint (proximal versus distal).

Based on these factors, different cyanoacrylate–ethiodized oil ratios can be used to achieve the same interventional goal, allowing a flexible approach with a range of dilutions rather than fixed values (Table 5) [9–49]. The minimum dilution should not be lower than 1:1 (cyanoacrylate:ethiodized oil) to avoid inadequate radiopacity and excessively rapid polymerisation before reaching the target. Conversely, the maximum dilution should not exceed 1:8, as excessive ethiodized oil reduces the embolic effect of cyanoacrylate.

**Table 5:** Cyanoacrylate to ethiodized oil dilution ratios according to literature data and suggested by the working group in standard conditions of blood flow and vascular anatomy

Fields of embolization	Dilution ranges reported in literature	Dilution ranges suggested by the SoP WG (in standard conditions)
Active haemorrhage (excluding GI)	1:1 – 1:9 [9–15]	1:2 – 1:4
Non-variceal GI haemorrhage	1:1 – 1:9 [16–19]	1:2 – 1:4
Tumour	1:2 – 1:6 [20–22]	1:2 – 1:4
PAE (benign prostatic hyperplasia)	1:6 – 1:10 [23–25]	1:6 – 1:8
BAE	1:1 – 1:14 [26, 27]	1:2 – 1:4
PVE	1:1 – 1:10 [28–33]	1:6 – 1:8
Pseudoaneurysms	1:1 – 1:3 [34–37]	DP:1:1 – 1:2 TC: 1:1.5 – 1:3
Varicoceles	1:1 – 1:1.2 [38, 39]	1:1
PCS	1:1 – 1:5 [40, 41]	1:1 – 1:3
AVMs	1:1 – 1:6 [42–45]	1:3 – 1:5
EL-2	1:1 – 1:10 [46–49]	DP: 1:1 – 1:2 TC: 1:2 – 1:4

GI: Gastrointestinal; PAE: Prostate artery embolization; BAE: Bronchial artery embolization; PVE: Portal vein embolization; PCS: Pelvic congestion syndrome; AVMs: Arteriovenous malformations; EL-2: Type 2 endoleak; SoP WG: Standards of Practice working group; DP: Direct puncture; TC: Transcatheter

Embolic preparation should be performed on a separate trolley to prevent contact with ionic solutions such as blood or saline, which can trigger premature polymerisation. Basic equipment includes two 5–10-mL Luer-lock syringes and a plastic three-way stopcock for cyanoacrylate dilution. Luer-slip syringes should be avoided during this stage of embolic preparation to minimise splash risk. After drawing up cyanoacrylate and ethiodized oil in the chosen ratio, gentle mixing through the stopcock, typically 4–5 transfers, produces a clear solution. If the mixture turns opaque, polymerisation has begun and the mixing process must be restarted [16, 35, 39].

#### Polymers

When preparing in situ–precipitating liquid embolics, the specific characteristics of each product must be considered, including the polymer solvent and the method of radiopacity, as these determine both patient preparation and product handling.

DMSO-based polymers are safe when injected slowly with a maximum recommended dose of 200 mg/kg [50]; however, DMSO can cause irritation and pain similar to ethanol; therefore, patients must be appropriately sedated or under general anaesthesia.

For EVOH preparation, vials must be placed on a mechanical agitator to resuspend the Tantalum microparticles that provide radiopacity; approximately 20 min of agitation is required. It is standard practice to begin agitation when the patient enters the angiography suite; unused, shaken EVOH can be returned to the shelf without degradation. Other polymers, such as PHIL® (Terumo Neuro, US), contain Iodine for radiopacity and therefore require no mixing [51].

Polymer viscosity selection follows the same patient- and operator-dependent considerations used for cyanoacrylate dilution. Polymer vials must not be diluted, as this alters stability. A general rule applies:

- High-viscosity polymers (e.g. Onyx®34, Squid®34, PHIL®35%) for proximal embolization
- Low-viscosity polymers (e.g. Onyx®18, Squid®12/18, PHIL®25–30%) for distal embolization.

### Injection Technique

#### Cyanoacrylates

Before cyanoacrylate injection, the microcatheter dead space must be primed with dextrose (5% or 10%) to prevent intraluminal blockage. A homogeneous glue cast should be obtained to avoid fragmentation and prevent consequent non-target embolization and ischaemia of healthy tissues. Three main approaches are used (Table 6): free-flow, blocked-flow, and percutaneous direct puncture (DP), with selection based on anatomy, flow, and procedural goals.

- Free-flow injection via microcatheter delivers a cyanoacrylate—ethiodized oil mixture without proximal occlusion, allowing forward blood flow to carry the embolic distally. This enables deep penetration into small or tortuous vessels but increases the risk of cast fragmentation, distal migration, and non-target embolization in high-flow settings [17, 52].
- Blocked-flow injection via microcatheter relies on temporary cessation of blood flow, achieved by using a balloon occlusion catheter or by wedging the microcatheter tip. This technique permits a more uniform polymerisation and controlled cast formation. Experimental studies show this approach produces denser casts with less reflux and reduced intimal injury [5]. The ‘plug-and-push’ variant creates a small proximal glue plug around the microcatheter tip, limiting reflux and blood flow, enabling subsequent injections to advance distally in a controlled manner [5]. This technique is analogous to that used with EVOH-based agents. It can be safely employed with cyanoacrylates exhibiting intermediate or low polymerisation rates, such as Glubran®2 (GEM, Italy) or MagicGlue® (Balt, France).
- Percutaneous DP involves injecting glue directly into a vascular lesion (e.g., pseudoaneurysm or arteriovenous malformation (AVM)) via a needle under US or fluoroscopic guidance, achieving localised occlusion while avoiding systemic embolization [35, 36].

**Table 6:** Injection techniques of cyanoacrylate glue for embolization

Technique	Description	Advantages	Disadvantages/risks	Typical dilution (cyanoacrylate:ethiodized oil)	Typical indications
Free-flow	Injection without proximal occlusion; relies on blood flow to carry glue distally	Good distal penetration; useful in tortuous/small vessels	Risk of distal non-target embolization; Cast fragmentation; Catheter entrapment if reflux occurs	1:3–1:8 (slower polymerisation)	AVMs, hypervascular tumours, PAE, small vessel embolization
Blocked-flow	Balloon or wedged catheter temporarily halts blood flow during injection	Compact, cohesive casts; Reduced migration risk; Better control	More risk of catheter entrapment; Requires specialised catheter/balloon (except plug and push technique)	1:1–1:3 (faster polymerisation)	High-flow lesions, pseudoaneurysms, GI bleeding, Gastro-oesophageal varices
Percutaneous	Direct needle puncture into lesion under imaging guidance (US/Fluoro/CT)	Highly targeted occlusion; Avoids systemic embolization; Minimally invasive in selected cases	Requires precise needle placement; Risk of extravasation or tissue necrosis	Variable, often 1:1–1:3	Pseudoaneurysms, superficial AVMs, tumours

US: Ultrasound; CT: Computed tomography; AVMs: Arteriovenous malformations; GI: Gastrointestinal; PAE: Prostate artery embolization

For injection through the microcatheter, 1-mL, 3-mL, or 5-mL Luer-lock syringes may be used depending on catheter size. With >2 Fr microcatheters, 3–5-mL syringes generally provide smoother injection with less resistance; for smaller-calibre microcatheters, a 1-mL syringe offers better control. Performing test injections with iodinated contrast using a syringe that is identical to the one then used for glue will enable the operator to estimate the volume to deliver and the injection speed.

After injection, the microcatheter can be reused if promptly flushed with dextrose or ethiodized oil; otherwise, a new microcatheter should be used.

An equally important technical variable is the dilution ratio of cyanoacrylate with ethiodized oil, which determines both polymerisation speed and cast morphology. More diluted mixtures (1:4–1:6) slow polymerisation, enabling deeper penetration but increase the risks of distal or non-target embolization [5]. More concentrated mixtures (1:1–1:2) polymerise rapidly, promoting proximal occlusion and minimising unintended spread [4]. Experimental evidence confirms that glue dilution and injection rate profoundly affect both radiologic outcomes and histologic vessel responses [4, 5, 8].

Finally, operators should also consider the volume of preparatory dextrose injected: distal embolization targets require generous dextrose flushing to minimise contact between cyanoacrylate and blood anions, favouring glue propagation into small vessels; proximal embolization requires only microcatheter dead space flushing, aiming to obtain early polymerisation just after injection. In all cases, the interval between dextrose and glue injection should be kept as short as possible and back-flow of blood in the microcatheter should be prevented [3, 18, 24, 45].

#### Polymers

DMSO-compatible microcatheters are mandatory for all polymer embolic agents [50, 51]. Specialised microcatheters with double distal radiopaque markers are available. These are particularly useful for monitoring peri-catheter reflux, an essential step during in situ precipitating polymer injections.

Before injection, the microcatheter lumen and hub must be thoroughly flushed with saline to eliminate any blood remnants that could prematurely trigger polymer precipitation. Luer-lock syringes are recommended and should remain attached after flushing to prevent blood reflux during product preparation. The microcatheter dead space is then filled with DMSO, after which polymer injection may begin. DMSO for catheter flushing should be injected as slowly as the following EVOH, because DMSO can cause severe vessel spasm, limiting the subsequent depth of EVOH penetration into the periphery [50].

**Fig. 5:**
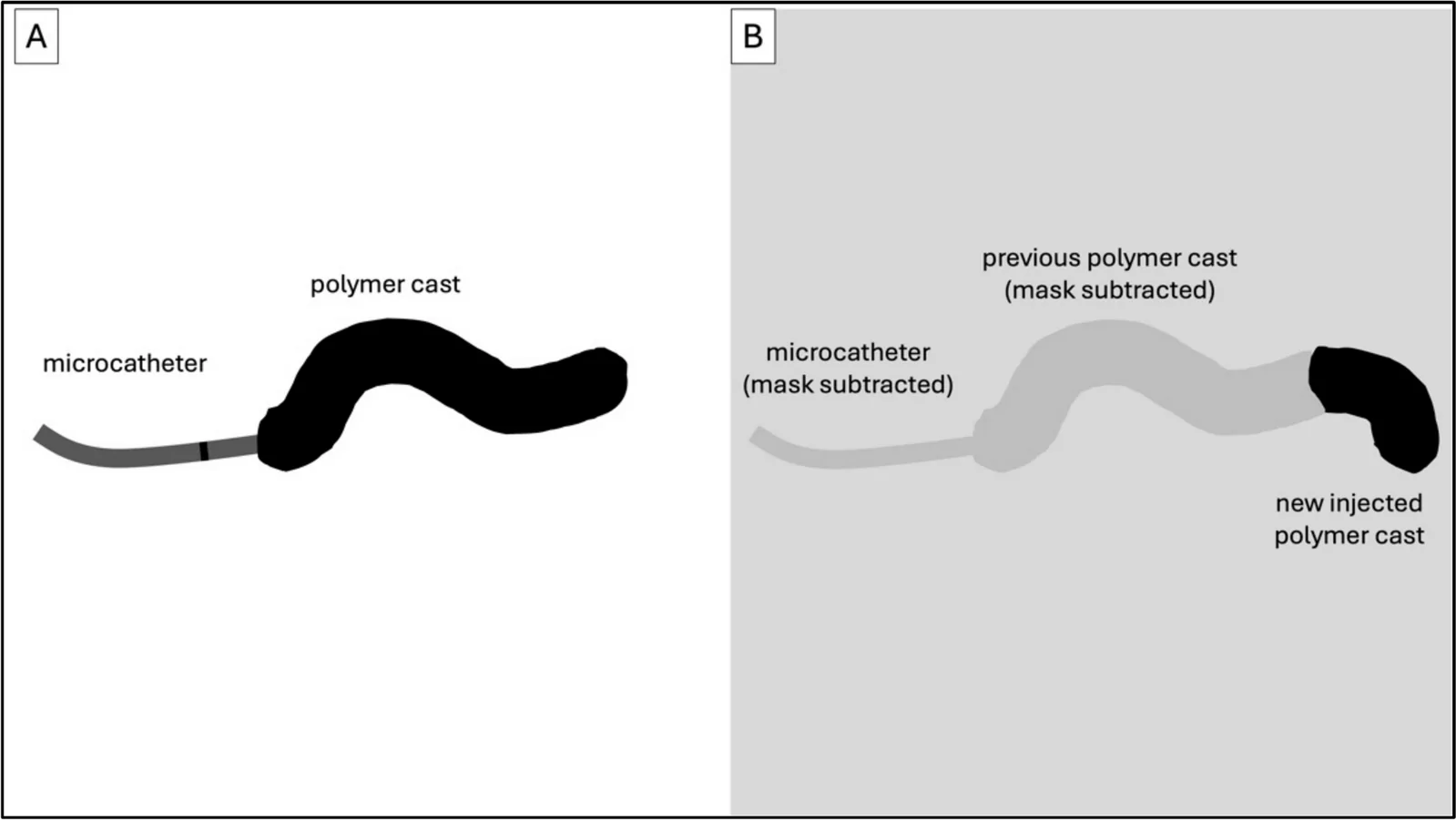
Reverse-roadmap technique during polymer injection. **A** Under standard fluoroscopic visualisation, via a microcatheter with double distal radiopaque markers, the first polymer injection is performed with reflux around the microcatheter tip (marker on the tip is into the cast); **B** After the 2 min required for cast solidification, a subsequent polymer injection is performed after acquiring a plain roadmap acquisition, the microcatheter and the first cast are subtracted by the roadmap mask and only the newly injected polymer appears radiopaque

Polymer delivery requires continuous fluoroscopic monitoring and a very slow injection rate (0.1–0.3 mL/min). Contact with blood produces a proximal ‘plug’ at the microcatheter tip, anchoring the cast and limiting reflux. After allowing 1–2 min for plug formation, the operator proceeds with slow, intermittent injections to achieve controlled antegrade progression into the target. As polymer embolization procedures can be prolonged, differentiating newly injected polymer from the existing cast may become challenging. In such cases, the reverse-roadmap technique is useful: acquiring a plain roadmap masks the catheter and earlier cast, facilitating visualisation of the newly injected polymer (Fig. 5). If reflux occurs, injection should be paused to allow solidification before resuming with a plug-and-push technique. Once the embolization endpoint is achieved, a 1–2 min pause allows the cast to harden. The microcatheter is then withdrawn carefully, ensuring that the tip remains free of polymer. Detachable-tip microcatheters may be considered for prolonged procedures or tortuous anatomy to reduce the risk of catheter entrapment [50–52].

Direct percutaneous polymer injection is also effective for otherwise inaccessible targets, such as aneurysm sacs or distal AVMs [35, 36, 53]. Lesions are punctured with an 18–22 G needle under imaging guidance (US, fluoroscopy, or CT/Cone-Beam CT). Correct needle placement is confirmed by slow blood reflux and, when required, contrast injection or parenchymography. Parenchymography is particularly useful for verifying that the neovascular compartment of a lesion has been accurately targeted. After priming the needle dead space with DMSO, the polymer is injected slowly under continuous reverse-roadmap visualisation; typically the tap technique is adopted and consists of making small taps at the end of the Luer-lock syringe with the operator’s thumb to inject at a rate of 0.1–0.2 mL/min, ensuring optimal control. Injections are repeated until adequate embolization is achieved [53].

### Combining NSLEA and Non-Liquid Embolics

The mechanism of action of NSLEA differs fundamentally from that of metallic and particulate agents, resulting in distinct advantages and limitations for each class. Consequently, the combined use of different embolic materials can be complementary, enhancing clinical effectiveness. In clinical practice, it is common to employ multiple embolic types within the same target vessel to overcome the inherent limitations of any single agent.

**Fig. 6:**
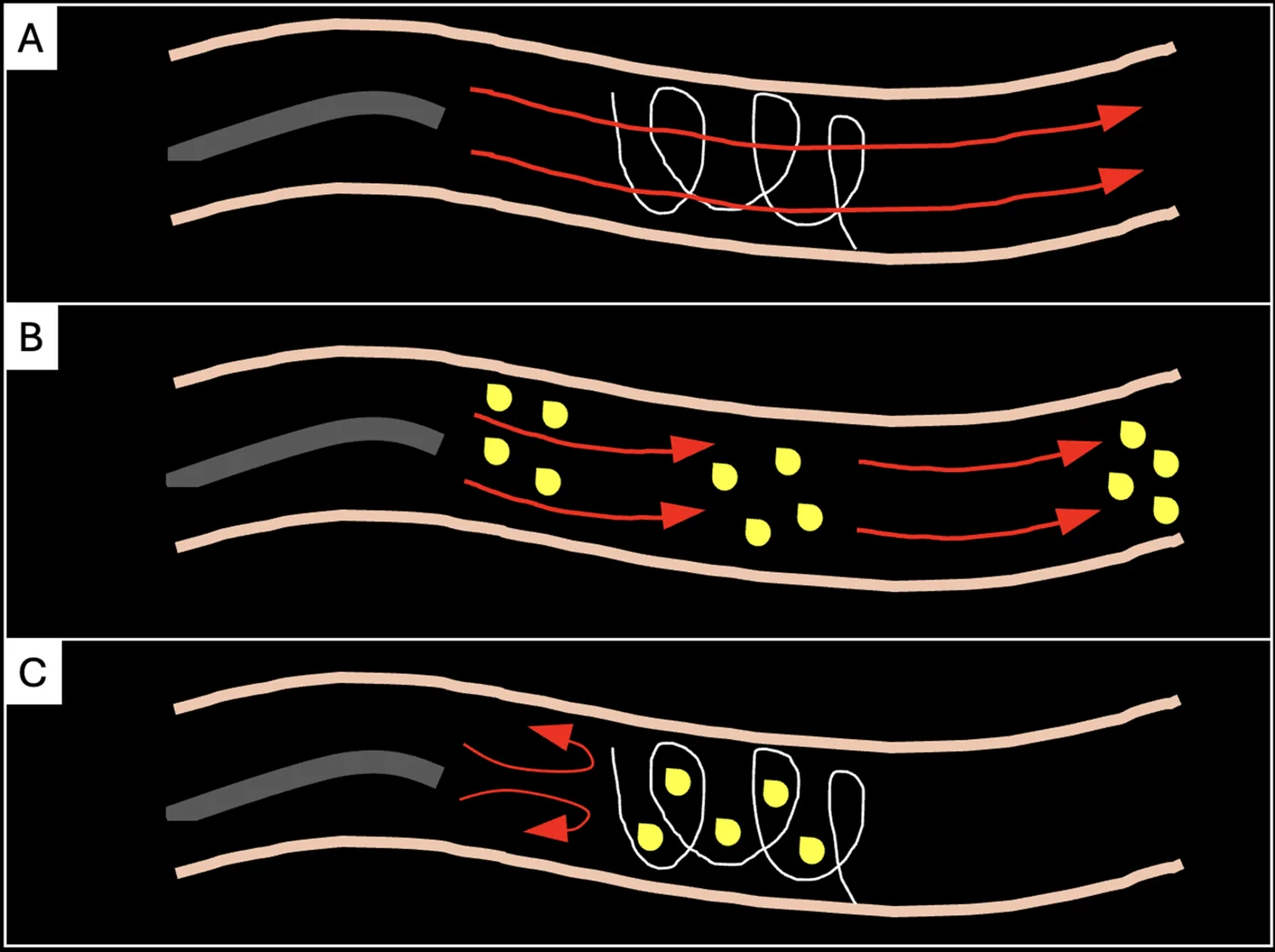
Combined approach of coils and NSLEA in a large vessel with coagulation anomalies. **A** Coils alone would not be able to block blood flow because of impaired coagulation. **B** NSLEA alone would migrate distally resulting in non-target complications. **C** Releasing a coil creates a scaffold for subsequent NSLEA entrapment; NSLEA droplets polymerise into the coil cast, allowing no distal migration of the liquid agent and resulting in blood flow occlusion

NSLEA are particularly well suited for small- to medium-sized vessels; however, in large vessels with high-flow velocities, there is an increased risk of distal migration and non-target embolization for both cyanoacrylates and polymers. In such scenarios, mechanical occluders are often used first to slow blood flow and provide a scaffold before completing the embolization with a NSLEA (Fig. 6) [54].

The combination of mechanical and NSLEA has been shown to be highly effective, achieving immediate and durable haemostasis in large vessels and in patients with coagulopathies [55]. The biocompatibility of both cyanoacrylates and polymers is high, and no adverse effects have been reported when these agents are combined with each other or with coils and plugs [56].

It is essential to emphasise that combination embolization should only be performed by experienced IRs, due to the dynamic changes in blood flow that can occur during delivery. Careful procedural planning is critical. For example, when using a single catheter for a combination approach, NSLEA should generally be delivered as the final step, as even trace amounts adhering to the inner lumen can compromise subsequent embolic delivery.

## Clinical Applications

Both cyanoacrylates and polymers are highly versatile embolic agents with a broad spectrum of clinical applications, and their use has increased steadily over the past decade (Table 7) [57–80]. Considering that, unlike mechanical or particulate embolics, NSLEA are effective in patients with impaired coagulation, these are particularly valuable in emergency settings or when temporary cessation of antiplatelet/anticoagulation therapies is not feasible [9–67].

**Table 7:** Clinical applications of liquid embolics in interventional radiological interventions

Fields of embolization	Cyanoacrylates	Polymers
Active haemorrhage (excluded GI)	+++ [9–15]	+ [55–58]
AVMs	+ [42, 44, 45]	+++ [7, 51, 59–61]
BAE	++ [26, 27, 62]	+ [63]
EL-2	+ [47–49]	+++ [56, 64, 65]
Non-variceal GI haemorrhage	++ [17–19]	+ [66, 67]
PAE (benign prostatic hyperplasia)	+++ [23–25, 68, 69]	+ [70, 71]
PCS	+ [40, 41]	+ [72, 73]
Pseudoaneurysms	++ [34–37]	+ [74]
PVE	+++ [28–32, 75]	+ [76, 77]
Tumour	+ [20–22]	+ [51, 58]
Varicoceles	++ [38, 39, 78]	–
Visceral aneurysms	+ [79]	+ [80]

GI: Gastrointestinal; AVMs: Arteriovenous malformations; BAE: Bronchial artery embolization; EL-2: Type 2 endoleak; PAE: Prostate artery embolization; PCS: Pelvic congestion syndrome; PVE: Portal vein embolization

^*^ **+++**: strong/high/well-established evidence; **++**: moderate evidence; **+**: weak or limited evidence; **–**: no evidence

The clinical indications for both types of NSLEA overlap and include the management of active bleeding [9–15, 17–19, 34–37, 55–58, 66, 67, 74], AVMs [7, 42, 44, 45, 51, 59–61], type 2 endoleaks (EL-2) [47–49, 56, 64, 65], prostate artery embolization (PAE) [23–25, 68–71], pelvic congestion syndrome (PCS) [40, 41, 72, 73], portal vein embolization (PVE) [28–32, 75–77], as well as preoperative or palliative tumour [20–22, 51, 58] interventions and visceral aneurysms [79, 80]; however, there are notable distinctions: polymers are generally not employed for male varicocele, whereas cyanoacrylates are suitable for this indication [38, 39, 78]; in practice, cyanoacrylates are more frequently used in emergency interventions, PAE, male varicocele, and PVE, whereas polymers are preferred for elective procedures, particularly in AVMs and EL-2 [9–80].

Anatomic and haemodynamic considerations also influence agent selection. In situations where there is a high risk of distal migration or reflux that could result in non-target embolization, controlled-release polymers are generally favoured. Conversely, the ability to adjust cyanoacrylate dilution allows operators to modulate penetration depth, providing a tailored embolization according to procedural goals. Ultimately, the choice between cyanoacrylates and polymers is flexible and should be guided by the operator’s experience, clinical context, and available resources.

## Patient Management

### Patient Preparation

No specific patient preparation is required for embolization interventions with NSLEA. In elective scenarios, standard laboratory parameters should be checked preprocedurally including: blood count, coagulation, renal and liver function. Antiplatelets and anticoagulation therapies must be suspended or managed according to international guidelines [81]. Recent contrast-enhanced imaging (CT or MRI) is needed to properly plan the procedural strategy in terms of vascular anatomy. In the case of a direct percutaneous approach [35, 53], an US evaluation of the target is useful to identify an appropriate safe acoustic window. Anaesthesiology evaluation should be conducted as well, together with appropriate specialist consultation in cases of identified comorbidities.

During preprocedural consultation in the Interventional Radiology Clinic [82], patients should be informed about possible complications related to liquid embolics in the specific intervention and a written informed consent should include these aspects. If polymers are used, the patient should be warned regarding DMSO factors, e.g. local inflammatory pain during injection and smelling like garlic for up to 48 h after the intervention. They should also be advised to warn other radiology departments about the dense artefact arising from the Tantalum when they attend for CT imaging.

In the case of emergency embolization, the above-mentioned assessments may not be feasible according to the clinical conditions.

### Procedural Medications and Post-Treatment Care

Drugs and therapies depend on the underlying disease and on the anatomical district rather than on the NSLEA.

Many elective embolization procedures (e.g. varicocele, PCS, PAE, etc.) can be performed without anaesthesiology support; however, sometimes deep sedation or general anaesthesia might be required according to clinical need. Furthermore, operators should be aware that in case of polymer injection, DMSO provokes immediate strong tissue inflammation [7, 50, 51, 58, 59]: therefore, painkillers and sedation should be considered before their use.

During the intervention, vital parameters, including heart rate (HR), peripheral oxygen saturation (SpO2), respiratory rate (RR) and arterial blood pressure (BP), are monitored and therefore must be maintained in standard ranges.

Post-embolization syndrome (PES) occurrence may be an expected consequence rather than a complication when liquid agents are selected [7, 34, 35, 38, 50, 51]; it can be managed with anti-inflammatory drugs and hydration support. If the embolization involves superficial structures, topical ice application can help in relieving local inflammations.

Endovascular embolization is categorised as a clean intervention [83], and therefore, antibiotic prophylaxis is not always required; however, depending on the district embolized, antibiotics can be administered to reduce the risk of abscess formation. No long-term medications are specifically needed in case of NSLEA application.

## Outcomes

NSLEA can be used in multiple clinical scenarios with high technical and clinical success rates. Haemorrhage [9–15, 17–19, 56, 57, 66, 67], AVMs [7, 42–44, 51, 60, 61], PAE [23–25, 68, 70], PVE [28–31, 33, 75–77] and varicoceles [38, 39, 78] are among the most common pathological conditions which are embolized with liquids (Table 8). In AVMs, the success rates present a wide percentage range (50–100%) because these are influenced by the complexity of the vascular network, requiring frequently multiple procedural approaches; in other fields, the high technical success rate underlines the effectiveness of the embolizing properties of NSLEA leading to clinical success. The mortality rate is overall low, depending mainly on the underlying condition, as in haemorrhage, rather than on the NSLEA itself.

**Table 8:** Technical success, clinical success and 30-day mortality descriptive ranges of the most common conditions for which liquid embolics are commonly used

Clinical condition	Technical success	Clinical success	30-day mortality
Active haemorrhage (excluding GI)	97.6–100% [9–15, 56, 57]	65.5–100% [9–15, 56, 57]	0–44% [9–12, 14, 15, 57]
AVMs	50–100% [7, 42–44, 51, 60, 61]	56.2–100% [7, 43, 44, 51, 60, 61]	0% [7, 42–44, 51, 60, 61]
Non-variceal GI haemorrhage	93.5–100% [17–19, 66, 67]	63–100% [17–19, 66, 67]	0–23.6% [17, 19, 66, 67]
PAE (benign prostatic hyperplasia)	100% [23–25, 68, 70]	86.4–100% [23–25, 68, 70]	0% [23–25, 68, 70]
PVE	97.2–100% [28–31, 33, 75–77]	62–87% [28–31, 33, 75–77]	0% [28–31, 33, 75–77]
Varicoceles	86.1–100% [38, 39, 78]	68.4–100% [38, 39, 78]	0% [38, 39, 78]

GI: Gastrointestinal; AVMs: Arteriovenous malformations; PAE: Prostate artery embolization; PVE: Portal vein embolization

## Specific Complications Relating to NSLEA

NSLEA, although highly effective across a broad range of clinical scenarios, are associated with a characteristic spectrum of potential complications. Their clinical significance depends on the vascular territory targeted and on the patient’s performance status; indeed, the full range of CIRSE-classified complications may occur—from minor, asymptomatic findings (grade I) to rare but severe adverse outcomes (grade V) [84]. Unlike mechanical or particulate embolics, NSLEA require operators to assess multiple interdependent variables—microcatheter position, haemodynamics, flow dynamics, collateral pathways, and the anticipated direction and extent of cast progression, before injection; so, formal training is essential. Importantly, NSLEA effects are permanent and largely irreversible.

Complications may occur during or after the procedure and are described with both cyanoacrylates and polymer-based agents (Table 9). Microcatheter entrapment [85, 86], although often cited, is rare with modern hydrophilic-coated microcatheters and approved formulations. Avoidance of excessive reflux, prevention of catheter redundancy, and appropriate flushing techniques further reduce this risk. Non-target embolization [9, 34, 35, 87, 88] remains the most frequent adverse event; when healthy tissues are inadvertently occluded, the resulting ischaemia can have minor to significant clinical consequences depending on the vascular territory involved [85, 89]. This risk is especially pronounced when highly diluted cyanoacrylate-ethiodized oil mixtures are used, given their distal penetration.

**Table 9:** Complications related to cyanoacrylates and polymers

Complication	Cyanoacrylates	Polymers
Microcatheter entrapment	+ [85]	+ [86]
Non-target embolization	++ [9, 34, 35]	+ [87, 88]
Unexpected proximal blocking	+ [85]	+ [86]
Tissue ischaemia	+ [85]	+ [89]
CT artefacts	–	+++ [7, 56, 64]
PES	+++ [34, 35, 38]	+++ [7, 51, 58, 91]
Vascular spasm	–	+ [50]
Acute respiratory distress	–	+ [90]
Tattoo effect	–	+ [60]

CT: Computed tomography; PES: Post-embolization syndrome. +: A rare complication, only anecdotal cases have been reported; ++: ≤ 5% of cases; +++: ≥ 15% of cases

Unexpected proximal catheter or microcatheter occlusion [85, 86] may occur with concentrated cyanoacrylates or polymeric agents, resulting in loss of superselective access. In cases of non-target embolization, a mechanical catheter-directed thrombectomy could be tried, although this is very often unsuccessful, because NSLEA stick to the vessel wall and are very flexible. In large vessels, trapping the agent against the vessel wall with stent(s) could be an option.

Additional considerations apply to polymers: Tantalum-based formulations may result in permanent cutaneous tattooing [60] when used in superficial territories and generate metallic artefacts on follow-up CT [7, 56, 64]. DMSO, when injected too rapidly or in excessive volume, may provoke substantial vasospasm [50] or acute cardiopulmonary reactions [90], compromising the intended embolic endpoint and, in extreme circumstances, becoming life-threatening.

Overall, most complications associated with NSLEA are minor (CIRSE grades I–II). PES characterised by mild pain, transient fever, and discomfort due to the expected local inflammatory response is common and should be anticipated as a consequence of the procedure, rather than a complication [7, 34, 35, 38, 51, 58, 91]. Its clinical impact varies by organ targeted but it is generally self-limiting or manageable with simple analgesia and anti-inflammatory therapy. Nonetheless, awareness of the potential for major events (CIRSE grades III–V) underscores the importance of meticulous adherence to preparation and injection protocols, together with rigorous patient selection.

A thorough understanding of these potential adverse events, together with adherence to recommended technical protocols, careful planning, and appropriate patient counselling, is fundamental to maximising the safety and efficacy of NSLEA procedures. A dedicated quality assurance process, based on local facilities together with materials and technique standardisation, could help IRs oversee complications occurrence.

## Conclusions

NSLEA are highly versatile, enabling superselective embolization of small and medium vessels across a wide range of clinical scenarios. Cyanoacrylates are generally preferred in emergencies, while polymers are favoured in elective procedures, though choice is largely guided by operator experience and preference. Combining liquid agents with other embolics further broadens their clinical applications.

Interest in NSLEA is growing among clinicians and manufacturers. Several new agents are expected in Europe, including polymers with radiopacity that diminishes over time, reducing CT metallic artefacts [92] and conformable embolics that adjust penetration according to shear stress, offering improved delivery control [93].

When used according to published technical guidelines, NSLEA are safe and effective. However, they require a longer learning curve than mechanical or particulate agents to achieve proficiency; dedicated IR training is essential to master microcatheters, needles and NSLEA and at the same time understand the flow dynamics of the target vascular territory. Given their unique properties, all IRs should be familiar with NSLEA and apply them flexibly, adhering to established recommendations.

## Abbreviations

AHCA: Alpha-hexyl cyanoacrylate
AVM: Arteriovenous malformation
BAE: Bronchial artery embolization
BP: Blood pressure
BPH: Benign prostatic hyperplasia
CIRSE: Cardiovascular and Interventional Radiology Society of Europe
CT: Computed tomography
DMSO: Dimethyl sulfoxide
DP: Direct puncture
EL-2: Type 2 endoleak
EVOH: Ethylene vinyl alcohol
GI: Gastrointestinal
HR: Heart rate
IRs: Interventional radiologists
MS: Methacryloxysulfolane
NBCA: N-butyl cyanoacrylate
NSLEA: Non-sclerosant liquid embolic agents
PAE: Prostate artery embolization
PCS: Pelvic congestion syndrome
PES: Post-embolization syndrome
pHEMA: Polyhydroxyethylmethacrylate
PVE: Portal vein embolization
RR: Respiratory rate
SoP WG: Standards of Practice Working Group
SpO2: Peripheral oxygen saturation
TC: Transcatheter
